# Different response to vitamin D supplementation in children with RVOT morphology PVCs vs LV fascicular PVCs

**DOI:** 10.1097/MD.0000000000047888

**Published:** 2026-03-13

**Authors:** Marius Bichescu, Simona Sorana Cainap, Cecilia Lazea, Daniela Iacob, Alina Negru, Gabriel Cismaru

**Affiliations:** a 5th Department of Internal Medicine, Cardiology Rehabilitation, “Iuliu Hatieganu” University of Medicine and Pharmacy, Cluj-Napoca, Romania; b Department of Pediatrics II, Emergency Clinic Hospital for Children, “Iuliu Hatieganu” University of Medicine and Pharmacy, Cluj-Napoca, Romania; c Department of Pediatrics I, Emergency Clinic Hospital for Children, “Iuliu Hatieganu” University of Medicine and Pharmacy, Cluj-Napoca, Romania; d Department of Pediatrics III, Emergency Clinic Hospital for Children, “Iuliu Hatieganu” University of Medicine and Pharmacy, Cluj-Napoca, Romania; e Department of Cardiology, “Victor Babeș” University of Medicine and Pharmacy, Timișoara, Romania.

**Keywords:** arrhythmias, pediatric, premature ventricular contractions, vitamin D

## Abstract

Outflow tract premature ventricular contractions (PVCs) are typically benign arrhythmias in structurally normal hearts. Vitamin D insufficiency has been shown to cause PVCs in both children and adults. This study examined the impact of vitamin D supplementation in children with an increased burden of PVCs in 2 patient categories with distinct electrocardiogram morphologies: right ventricular outflow tract (RVOT) and left fascicular. We enrolled 46 patients (mean age 10.6 ± 4.1 years, 26% female) showing an increased burden of monomorphic PVCs (mean = 18,925/24 hours) and vitamin D deficiency. We compared 36 patients with RVOT morphology and 10 patients with left ventricular fascicular morphology. In the RVOT morphology group, the mean age was 12.7 ± 2.7 years, comprising 75% males and 25 females, with a PVC burden of 18,343.7 ± 13,836.2/24 hours and a 25-OH vitamin D level of 23.5 ± 9.4 ng/mL. After 2 months of oral vitamin D supplementation, the vitamin D level increased to 41.6 ± 6.3, followed by a considerable reduction in PVC burden to 3628.0 ± 2347.2. This group of children showed 80% reduction in PVC burden. In the left ventricular fascicular morphology group, the mean age was 6.9 ± 5.5 years, comprising 71% males and 29% females, with a PVC burden of 20,535.3 ± 20,867.9, and a vitamin D level of 25.8 ± 7.1 ng/mL. Following 3 months of oral vitamin D supplementation, vitamin D levels increased to 65.8 ± 42.8 ng/mL; however, there was no significant change in PVC burden (19,207.1 ± 22,807.8). Vitamin D supplementation may be effective in reducing PVC burden in children with vitamin D deficiency and RVOT PVCs.

## 1. Introduction

Premature ventricular contractions (PVCs) are common in the general population. The prognosis is dependent upon the presence of structural heart disease. Idiopathic PVCs originating from the right ventricular outflow tract (RVOT) are considered benign. Electrolyte imbalances, such as hypokalemia and hypomagnesemia, are recognized causes of RVOT PVCs.^[1,2]^ However, other metabolic abnormalities may also associate with an increased burden of PVCs In particular cases, understanding the reason behind the PVCs facilitates the treatment of the underlying cause which may avoid the need for antiarrhythmic drugs.^[3]^

The role of vitamin D in the cardiovascular system is increasingly evident, with recognized effects in patients with heart failure, acute myocardial infarction, metabolic syndrome^[4]^ and arrhythmias. An association has been demonstrated between vitamin D levels, calcium, and cardiac arrhythmias, including non-valvular atrial fibrillation, with case reports indicating a relationship between serum vitamin D concentration and ventricular arrhythmias. A patient with vitamin D deficiency and a high burden PVCs showed a positive response to vitamin D supplementation, leading to a substantial reduction in arrhythmia burden.^[5]^ The presence of PVCs in both twins with vitamin D deficiency indicates the influence of this disorder on arrhythmia genesis. PVCs disappeared with the correction of vitamin D deficiency in both twins.^[6]^ However, randomized trials investigating the reduction of PVC burden after vitamin D supplementation are absent.

This pilot study aimed to analyze the correlation between serum 25(OH)D levels and 2 morphological forms of PVCs: RVOT morphology and left ventricular (LV) fascicular morphology, and assess whether patients with monomorphic PVCs could benefit from vitamin D administration.

## 2. Materials and methods

### 2.1. Study subjects and procedure

A total of 46 patients from 3 Pediatric Clinics: Nos. 1 to 3 from Cluj-Napoca University Center were included in this study. They were evaluated in the Outpatient Clinic of the Rehabilitation Hospital Cluj-Napoca, Romania. All patients were children with a maximum age of 17 years. We excluded children with a structural heart disease. The study was approved by the Institutional Board of the Rehabilitation Hospital No. 1916/24.02.202 and patients gave their consent to use their de-identified data in the study. Individual participant data and other materials are stored on MEGA cloud and can be accessed through the following link: https://mega.nz/fm/UEwmGAqC. Parents signed the informed consent. Patients’characteristics are presented in Table 1. The PVC burden was assessed by 24-hour Holter electrocardiogram monitoring. All patients had high burden PVCs >5.000/24 hours. Vitamin D active metabolite concentration was assessed by sequenced blood samples. All patients had vitamin D deficiency <30 ng/mL.

**Table 1:** Comparison between RVOT and LV fascicular groups of premature ventricular contractions.

	RVOT (36)	LV fascicular (10)	
Male sex No (%)	27 (75%)	7 (70%)	0.201
Age (yr)	12.7 ± 2.7	6.9 ± 5.5	**0.007**
LVEF %	60	58	0.689
QRS duration (ms)	130.4 ± 13.0	89.1 ± 14.1	**<0.001**
QRS pattern V1	rS (32) QS (4)	rsR (4) rSR′(4) R (2)	–
QRS frontal axis	Inferior (36)	Inferior (2); superior (8)	–
Baseline 24 h PVCs No	18,343.7 ± 13,836.2	20,535.3 ± 20,867.9	0.702
Baseline 25-OH vitamin D ng/mL	23.5 ± 9.4	25.8 ± 7.1	0.228
2 mo 24 h PVCs	3628.0 ± 2347.2	19,207.1 ± 22,807.8	**0.047**
2 mo 25-OH vitamin D	41.6 ± 6.3	65.8 ± 42.8	0.170

Age, QRS duration and arrhythmia burden after 2 months of treatment are significantly different between the 2 groups (*P* < .05).

LV = left ventricular, LVEF = left ventricular ejection fraction, PVC = premature ventricular contraction, QRS = Q wave, R wave and S wave, RVOT = right ventricular outflow tract.

The intervention consisted of daily administration of 2000 to 4000 IU vitamin D and reassessment of both the number of PVCs and vitamin D concentration at 1 to 2 months after beginning of treatment. During the 2 months vitamin D treatment, no antiarrhythmic medication was given. Because of their lack of response to vitamin D treatment and high burden PVC, participants in the LV fascicular group received after completion of the study β-blockers (Propranolol) or class 1C antiarrhythmic medication (Flecainide, Propafenone).

### 2.2. Statistical analysis

All values were tested for normal distribution using the Shapiro–Wilk test. Continuous variables are presented as means ± standard deviation in case of normal distribution or median + interquartile range in case of non normal distribution. Categorical variables are presented as frequencies and percentages. Spearman correlation was used to assess association between the number of PVCs at baseline and 25-OH vitamin D levels as well as after vitamin D supplementation. All tests were performed using SPSS statistics program version 25 (IBM Corp., Armonk) and *P* values < .05 were considered statistically significant.

## 3. Results

Of the 46 patients included in the study, 34 were males and 12 females. Mean age was 10.6 ± 4.1 years old. Twenty-four hours Holter electrocardiogram revealed high burden monomorphic PVCs in both groups. Mean 25-OH vitamin D was <30 ng/mL in both groups.

We compared the 36 patients with RVOT morphology with 10 patients with LV fascicular morphology. RVOT morphology was characterized by left bundle branch block pattern, inferior frontal axis, and precordial transition V3–V4 and QRS duration 130.4 ± 13 ms. LV fascicular morphology was characterized by RBBB pattern in lead V1, superior axis (n = 8) or inferior axis (n = 2) and QRS duration of 89.1 ± 14 ms. The QRS duration was significantly narrower in the fascicular group (*P* = .012).

In the RVOT morphology group, mean age was 12.7 ± 2.7 years with 75% males and in the LV fascicular morphology group, patients were younger (mean age 6.9 ± 5.5 years), 70% being males.

There was no significant difference of PVC burden between patients with RVOT and LV fascicular morphology: PVC burden was 18,343.7 ± 13,836.2 on 24 hours in the RVOT group and 20,535.3 ± 20,867.9 on 24 hours in the LV fascicular group.

25-OH vitamin D levels were 23.5 ± 9.4 ng/mL in the RVOT group and 25.8 ± 7.1 ng/mL in the LV fascicular group without a significant difference between groups.

**Figure 1.**
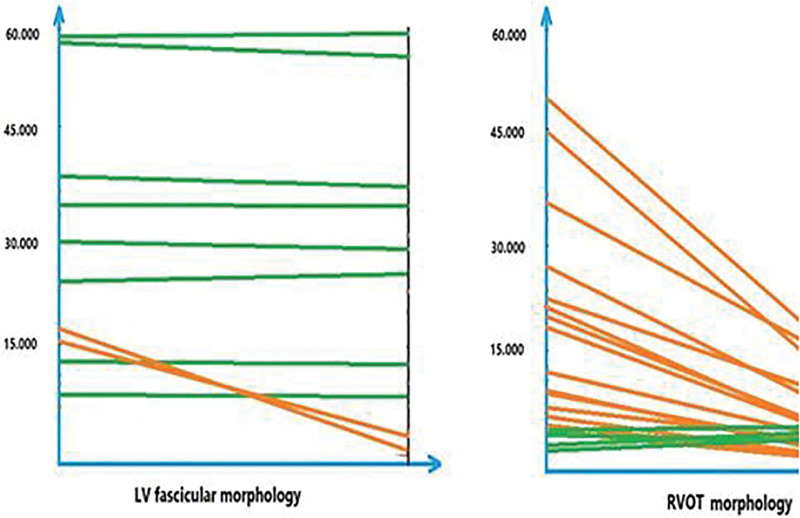
PVCs with an LV fascicular morphology have a poor therapeutic response to vitamin D supplementation. On the contrary, PVCs with an RVOT morphology have a good response to vitamin D supplementation. LV = left ventricular, PVC = premature ventricular contraction, RVOT = right ventricular outflow tract.

In the RVOT group, after 2 months of supplementation with oral vitamin D there was a significant increase in the level of vitamin D to 41.6 ± 6.3 ng/mL (*P* < .001) with significant decrease of PVC burden to 3628.0 ± 2347.2 on 24 hours (*P* < .001). Overall there was 80% decrease in PVC burden. However, in the LV fascicular group, after 2 months of supplementation with oral vitamin D, there was a significant increase in the level of vitamin D to 65.8 ± 42.8 ng/mL (*P* < .001) but without significant change in PVC burden 19,207.1 ± 22,807.8 on 24 hours (*P* = .235; Fig. 1).

## 4. Discussion

Our study was the first to explore the different response to vitamin D supplementation in 2 categories of PVCs: RVOT and LV fascicular PVCs. RVOT PVCs have a left bundle branch block morphology, with inferior axis, displaying a late precordial transition in lead V3–V4. In contrast, LV fascicular PVCs have a relatively narrow QRS with a sharp initial deflection as the Purkinje system is engaged. PVCs originating in the left posterior fascicle have a typical RBBB morphology with superior frontal plane axis, while PVCs from the anterior fascicle have a rightward axis.^[7,8]^ After 2 months vitamin D supplementation there was a significant increase in the 25-OH vitamin D levels in both groups, but with a significant decrease in the arrhythmic burden only in the RVOT group.

Different response to vitamin D supplementation can be explained by the differences between the working myocardium and the conduction system. The working myocardium has specific properties permitting enhanced automaticity and triggered activity, whereas the insulated by connective sheaths left fascicles have unique characteristics of enhanced automaticity.^[9]^ The outflow tract has different embriologic origin as it derives from the mesoderm located anterior namely the cephalic mesoderm. However, the left posterior and left anterior fascicles have a subendocardial origin which permits after full differentiation, cell-to-cell conduction and spontaneous electrical activity.^[10]^

Compared to RVOT PVCs, fascicular PVCs occur in younger children and usually completely disappear during childhood. However, RVOT PVCs, may last throughout adolescence and adulthood.^[11,12]^ We found a significant difference between the age in the 2 groups: patients with LV fascicular PVCs being younger than those with RVOT PVCs. This is consistent with the age of 10.5 ± 4.9 years reported by Collins et al on their study on 129 children with fascicular VT.^[13]^

Currently, multiple treatment strategies are available for arrhythmia management, ranging from no treatment, lifestyle changes, to vitamin and electrolyte supplements, use of antiarrhythmic drugs, or more invasive procedures like catheter ablation.^[14,15]^ Experimental studies and several reports demonstrated a beneficial effect of vitamin D in patients with PVCs.^[16,17]^ Animal models provide more and more insight into vitamin D’s role in cardiac muscle function. In vitro studies have shown that vitamin D acts directly on atrial and ventricular myocytes. Cardiac myocytes isolated from vitamin D receptor (VDR) knockout mice have rapid contraction rate, and vitamin D directly affects contractility in wild type mice but not in VDR knockout myocytes. This evidence suggests that vitamin D and VDRs play a role in heart contractility and abnormal excitability.^[18]^ Weishaar et al demonstrated that hypercontractility in isolated perfused hearts from rats with vitamin D deficiency, which might explain the occurrence of ventricular premature contractions.^[19]^ Green et al^[20]^ also showed the effect of vitamin D supplementation on isolated rat cardiomyocytes, with decrease of the contraction rate and increase in the relaxation rate. While the physiological significance of this effect is unknown, it demonstrates a rapid direct action of 1,25(OH)_2_D_3_ on cardiomyocytes that is dependent on the presence of the VDR and can result in PVCs.^[21]^

The mechanism behind the occurence of PVCs in patients with vitamin D deficiency may be related to increased calcium concentration in the myocyte, which will increase the amount of cyclic adenosine monophosphate present in the cell, resulting in the augmentation of calcium influx. Insufficient vitamin D concentration may lead to increase in cyclic adenosine monophosphate, proving its role in possible calcium induced PVCs. Even though the direct connection between vitamin D level and serum calcium has not been completely quantified, significantly reduced vitamin D levels lead to diminished intestinal calcium absorbtion and will reduce calcium-induced calcium release at myocyte level, which could produce calcium channel malfunction with membranary distrubances. Furthermore, vitamin D deficiency is associated with increased parathormone parathormone levels, which decreases cellular calcium intake and reduces calcium reuptake to the sarcoplasmic reticulum, and therefore increases intracellular calcium levels leading to exaggerated ventricular excitability.^[22]^

The presence of VDRs in over 30 human tissues, such as bone, cartilage, intestine, kidney, and even β-type pancreatic tissue, indicated the possibility of the expression of such a receptor at the level of the cardiac myocytes, as well as an important role in the function of the heart.^[23]^ In 1985, a cardiac receptor for the active metabolite of vitamin D was identified in the myocardial cell: the 1,25-dihydroxyvitamin D_3_ receptor.^[24]^ Chen et al^[25]^ used a combination of biochemical, immunofluorescence, and functional studies to demonstrate VDR in cardiac myocytes and fibroblasts, as well as ventricular myocardium. Calcitriol (1,25-dihydroxyvitamin D_3_) the active form of the hormone interacts with the specific VDR. The presence of VDR together with the vitamin D_3_-dependent calcium-binding protein regulate the action of vitamin D and calcium on cardiac metabolism both in hypocalcemic and normocalcemic states. The presence of a vitamin D-dependent calcium-binding protein may indicate a significant role for calcitriol on the physiological cardiac function,^[19]^ independent from serum calcium level.

The therapeutic option with vitamin D supplementation could be useful for patients with a high arrhythmic burden and might replace antiarrhythmic drugs or catheter ablation. Randomized trials are needed to confirm the findings of our preliminary study.

### 4.1. Limitations

The main limitation of the study is the low number of patients. However, this is a preliminary pilot study which will be followed by research on a higher number of patients. The 12 lead morphology of PVCs from the first group suggested ROVT origin. However, we cannot completely exclude left ventricular outflow tract origin for patients with V3 transition as we did not perform electroanatomical mapping.

## 5. Conclusions

This preliminary investigation suggests that vitamin D supplementation in patients with vitamin D deficiency may reduce the burden of RVOT PVCs but not the burden of LV fascicular PVCs. This may indicate a future therapeutic approach for patients with vitamin D deficiency and an increased burden of RVOT PVCs.

## Author contributions

**Conceptualization:** Gabriel Cismaru, Marius Bichescu.

**Data curation:** Gabriel Cismaru, Marius Bichescu, Simona Sorana Cainap, Cecilia Lazea, Daniela Iacob, Alina Negru.

**Formal analysis:** Gabriel Cismaru, Marius Bichescu.

**Funding acquisition:** Gabriel Cismaru, Simona Sorana Cainap.

**Investigation:** Gabriel Cismaru, Marius Bichescu, Simona Sorana Cainap, Daniela Iacob, Alina Negru.

**Methodology:** Gabriel Cismaru, Marius Bichescu.

**Project administration:** Gabriel Cismaru.

**Resources:** Gabriel Cismaru, Simona Sorana Cainap, Cecilia Lazea.

**Software:** Gabriel Cismaru, Marius Bichescu.

**Supervision:** Gabriel Cismaru, Simona Sorana Cainap, Cecilia Lazea.

**Validation:** Gabriel Cismaru, Marius Bichescu, Simona Sorana Cainap, Cecilia Lazea, Daniela Iacob, Alina Negru.

**Visualization:** Gabriel Cismaru, Marius Bichescu, Simona Sorana Cainap, Cecilia Lazea, Daniela Iacob, Alina Negru.

**Writing – original draft:** Gabriel Cismaru, Marius Bichescu.

**Writing – review & editing:** Gabriel Cismaru, Marius Bichescu, Simona Sorana Cainap, Cecilia Lazea, Daniela Iacob, Alina Negru.
